# Time trend analysis and demographic features of inflammatory bowel disease in Tehran 

**Published:** 2015

**Authors:** Hedieh Balaii, Hamid Asadzadeh Aghdaei, Alma Farnood, Manijeh Habibi, Amir Ali Mafi, Farzad Firouzi, Afsaneh Sharifian, Shabnam Shahrokh, Farhad Lahmi, Homayoun Zojaji, Nosratollah Naderi, Mohammad Reza Zali

**Affiliations:** 1 *Basic and Molecular Epidemiology of Gastrointestinal Disorders Research Center, Research Institute for Gastroenterology and Liver Diseases, Shahid Beheshti University of Medical Sciences, Tehran, Iran*; 2 *Gastroenterology and Liver Diseases Research Center, Research Institute for Gastroenterology and Liver Diseases, Shahid Beheshti University of Medical Sciences, Tehran, Iran*; 3 *AJA University of Medical Sciences, Tehran, Iran*

**Keywords:** IBD, Ulcerative colitis, Crohn’s disease, Iran

## Abstract

**Aim:**

This retrospective study is aimed to review demographic and clinical characteristics of IBD to elucidate the probable factors associating with IBD development in Taleghani Hospital in Iran since 2001 during a 12-year-period.

**Background:**

Ulcerative colitis (UC) and Crohn’s disease (CD) are two major idiopathic entities of inflammatory bowel disease (IBD). Previous studies have reported an increased incidence of IBD in Middle East countries.

**Patients and methods:**

In the present study 1914 patients with UC, 318 patients with CD and 25 with indeterminate colitis (IC) were included. Demographic information, clinical features, extraintestinal manifestations, complications and extension of disease were collected and interpreted for all participants. According to the time of registration, patients were divided into seven groups. Statistical analysis was performed using the chi-square test.

**Results:**

In seven groups of IBD patients, disease registry was estimated for UC, CD, and total IBD during a 12-year-period. From 2001 to 2005, a relative increased registry was observed among UC patients. However, in the years 2006 and 2007 a ​​significant reduction in the number of patients was reported. Then an increasing trend was observed in UC patients. UC presented mostly with diarrhea, hematochezia and bloody diarrhea, while most of CD patients complained of abdominal pain.

**Conclusion:**

Evaluation of data related to registered IBD patients in Iran shows that probable incidence and prevalence of IBD (UC and CD) is increasing compared to previous decades.

## Introduction

 Inflammatory bowel disease (IBD), including ulcerative colitis (UC), Crohn’s disease (CD) and another rare disorder with an intermediate features between ulcerative colitis and Crohn’s disease, which termed indeterminate colitis (IC), is a chronic and recurrent disease triggered by genetic, environmental, and immunologic factors (1).

IBD is reported more common in developed countries than developing countries. However, recently according to some studies, IBD prevalence is increasing in developing nations in the past two decades (2).

Different frequencies of IBD in male and female were reported (3). The diagnosis of IBD has two age peaks. In most populations the first peak is between 15 to 30 years and the second peak of IBD occurs between ages 50 to 70 (4). The major presenting symptoms in UC involving the colon, are bloody diarrhea, mucus in the stools, abdominal pain, and weight loss. The CD can affect any part of the digestive tract, however the terminal ileum is the commonest site for the disease. The CD clinically presents with abdominal pain, diarrhea and weight loss (5).

Extra intestinal manifestations (EIM), occurring in 25-40 percent of IBD patients, can be seen in any organ system, such as musculoskeletal, skin, hepatopancreatobiliary, ocular and renal systems. Musculoskeletal disorders have been detected as the most common EIM in IBD (6).

Due to the lack of a central data registry system in Iran, there have been few epidemiological studies and current time trends of IBD in Iran. Therefore, the incidence and prevalence remain unknown (-).

The aim of this study was to review the demographic features and clinical characteristics, extra-intestinal manifestations, complications, extension of disease and diagnosis identifications in outpatients and inpatients with IBD who referred to Taleghani hospital within a 12-year-period. 

## Patients and Methods

During a 12-year-period (between 2001 and 2013), 2257 patient’s data were recorded in a questionnaire, including demographic information, medical, family and habitual history, diagnosis, identification, signs and symptoms at onset as well as date of visit, extra intestinal manifestations, complications and colonoscopy reports at Taleghani Hospital, Tehran, Iran. 

The interview was performed face to face by a trained practitioner. General information was retrieved from medical records of patients or by a trained gastroenterologist. Moreover, the additional information gathered by telephone contact. IBD was confirmed by a gastroenterologist in patients based on diagnostic, clinical, radiological, endoscopic and pathological criteria, suggested by Lennard Jones (13).

Data was entered into an access database which was designed according to our questionnaire and updated through referring IBD patients for determining any other changes such as hospitalization, drug use, colonoscopy, pathology, laboratory tests or even new disease. 

According to the time of registration, patients were divided into 7 groups: 1) Registered before 2002, 2) 2002-2003, 3) 2004-2005, 4) 2006-2007, 5) 2008-2009, 6) 2010-2011 and 7) 2012-2013.

Descriptive age data of patients were presented as mean ± standard deviation. Comparison of background variables such as gender, breast feeding history, smoking and familial history were performed using Chi-square test and P-value <0.05 was considered as significant.

This study has been approved by the Ethics Committee of Shahid Beheshti Medical University, Tehran, Iran. 

## Results

From 2001 to 2013, 2257 patients with IBD were admitted to Taleghani hospital, Shahid Beheshti University. All patients were registered to an IBD data registry system including, 1914 patients with UC, 318 with CD and 25 with IC. 

**Figure 1:**
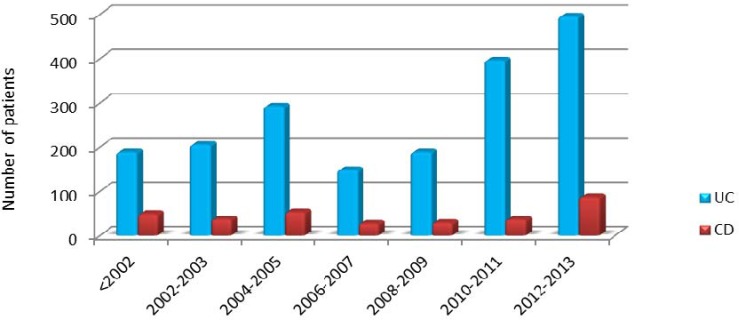
Time trend of registered IBD types in Taleghani hospital

The number of patients with IBD during a period of 2 years from 2001 to 2013 is shown (Figure 1). From 2002 to 2005, ulcerative colitis had a growing trend, and then declined steadily in 2006-2007. While in Crohn's disease there were slight variations in the number of patients over the years.

**Figure 2:**
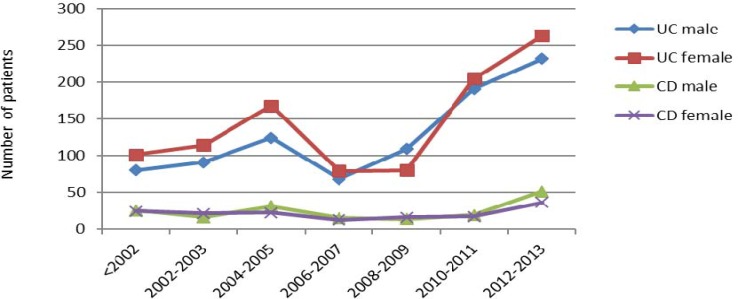
Sex distribution in IBD

 The male to female ratio for each group was pictured in figure 2. Ulcerative colitis had a significant increase among women than men. With the exception of the years 2008-2009, more men than women are affected by this disease. On the other hand, Crohn's disease is more common in men than women over these years. In two groups, in 2002-2003 and 2008-2009 outbreak was higher in women than in men.

The mean (±SD) age at diagnosis in each group is described (Table 1). Age at diagnosis of ulcerative colitis in ≤2001, 2008-2009 and 2012-2013 has not changed significantly, but 2004 – 2005 has a 1 year decrease. Ulcerative colitis from 2001 to 2009 was diagnosed almost one year after onset of symptoms. The lag time between onset of symptoms and age of diagnosis, in the recent years, has been decreased. The variance between the age of onset of Crohn's disease symptoms and diagnosis in 2001 and 2004-2005 was similar. This similarity in 2002-2003 and 2006-2009 could also be seen. Such as ulcerative colitis, Crohn's disease had a decreased the lag time in last 4 years (Table 2).

**Table 1:** The mean (±SD) age at diagnosis in each group

**Year**	**Ulcerative colitis**	**Crohn’s disease**
≤2001	33.16 ± 13.33	33.28 ± 12.65
2002-2003	33.19 ± 12.64	30.2 ± 13.37
2004-2005	32.63 ± 13.56	35.50 ± 13.50
2006-2007	34.27 ± 15.86	30.53 ± 12.36
2008-2009	33.41 ± 13.54	31.75 ± 14.27
2010-2011	35.17 ± 13.44	33.28 ± 12.50
2012-2013	33.87 ± 13.35	32.98 ± 15.66

**Table 2:** The mean lag time between age of onset and age at diagnosis

**Year**	**Ulcerative colitis**	**Crohn’s disease**
≤2001	1.6	2.2
2002-2003	1.4	1.8
2004-2005	1.4	2.2
2006-2007	1.1	1.6
2008-2009	1.2	1.9
2010-2011	0.9	0.9
2012-2013	0.7	0.7

**Figure 3:**
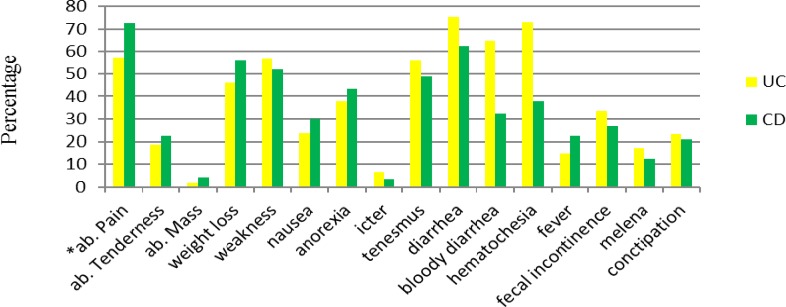
Chief complains of IBD patients

**Figure 4:**
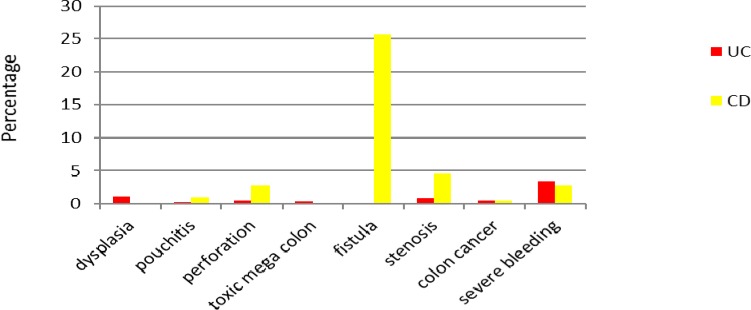
Major complications of IBD patients

**Table 3:** Demographic and clinical features of IBD patients

**Variables**		**UC**	**CD**	**p-value**
Sex				0.269
	Male	%47	%53	
	Female	%53	%47	
Breast feeding		%85	%85	0.810
Smoking				0.046
	Nonsmoker	%93.4	%88.7	
	Smoker	%3.9	%6.6	
Familial history		%14.2	%15.8	0.564
Positive familial history				0.483
	First degree	%57.9	%57.1	
	Second degree	%42.1	%42.9	
Colectomy		%2.8	%15.8	<0.001
Appendectomy		%4.4	%14.1	<0.001

**Table 4:** Extra intestinal manifestations in IBD patients

**Manifestations **	**IBD**	**UC (%)**	**CD (%)**	**p-value**
Eye Disorder	5.4%	4.9%	7.7%	0.270
Skin Disorder	18.2%	17%	23.4%	0.088
Musculoskeletal Disorder	26.8%	26%	30.1%	0.327
Liver & Biliary Disorder	8.1%	8.8%	5.1%	0.166
Urinary Tract Disorder	8.3%	8.3%	9.7%	0.240

Analyzing total data of the IBD registry in these years elucidated some clinical features of IBD in Iran. Table 3 shows the demographic and clinical features in IBD patients. In this study, 85% of UC and CD patients had a breast-feeding history during infancy. Among all patients, 93.4% patients with UC and 88.7% with CD had never smoked and 3.9% patients with UC and 6.6% with CD were current smokers. Familial history of IBD was presented in 14.2% UC patients, of whom 57.9% had a first-degree affected relative and 42.1% had second-degree affected relatives. IBD familial history was presented in 15.8% cases of CD patients, including 57.1% in first- and 42.9% in second-degree relatives. Totally, 2.8% of UC patients and 15.8% of CD patients underwent colectomy. Appendectomy was carried out in 4.4% UC patients and 14.1% CD patients. We found no significant difference between patients with UC and CD for gender, breast-feeding and familial history. However, the frequency of smoking (P= 0.046), colectomy (P< 0.001) and appendectomy (P< 0.001) were statistically significant higher among CD patients.

The major chief complaints of IBD were summarized in figure 3. UC presented mostly with diarrhea (74.3%), on the other hand, most of CD patients complained of abdominal pain (72.4%). 

The distribution of some symptoms defer statistically significant between IBD patients including abdominal pain and fever, which were more frequent in CD patients but diarrhea, bloody diarrhea, hematochezia and constipation had more frequency in UC patients. 

Extraintestinal manifestations were reported in 59.9% of UC patients and 60.2% of CD patients. Musculoskeletal (26.8%) and skin (18.2%) disorders were the most common affected sites in IBD patients. Also, 8.1% of UC patients were diagnosed with sclerosing cholangitis. Extraintestinal manifestations of IBD are shown in Table 4.

Major complications of UC were reviewed in this study (Figure 4), including dysplasia (1.1%), pouchitis (0.2%), intestinal perforation (0.5%), toxic mega colon (0.4%), stenosis (0.9%) and severe bleeding (3.4%). According to our finding, 25.6% of patients with CD had documented fistula. Colorectal cancer was determined in 1% of IBD patients (equally in UC and CD patients).

Extension of disease at diagnosis in UC patients was as follows: 30.8% proctitis, 40.2% left side colitis, and 28.9% pancolitis. Colonoscopy was performed in CD patients in which 71.8% were categorized as having ileocolitis and terminal ileum was affected in 21.8% of patients. 

## Discussion

In this present survey, the number of registered UC patients was 5 times higher than CD patients. Similar to the fourfold higher incidence of ulcerative colitis than Crohn's disease in Japanese (14), and Chinese cohorts (15). IBD is relatively rare in Asia, but it is more common in western countries, especially more prevalent in CD than UC (16). However, according to recent studies, both diseases have been reported with an increase in number in eastern countries (17). Generally, due to lifestyle changes, inflammatory bowel disease has an increasing trend in this study. Different clinical features of IBD in Iran from those in western countries might be due to the differences in their racial and geographic conditions. Therefore, analysis on the demographic, clinical characteristics, prevalence and incidence of patients with IBD may give some valuable clues to the diagnosis and management of the disease.

An equal gender distribution of CD in this data conflicted with Asian studies, which appear with a male-predominant (18). On the other hand, the demographic finding of UC in Iran shows a slight female predominant, but in western and Asian studies there is an equal sex distribution in UC.

In our study CD was diagnosed at a younger age than UC. The peak age of onset in Iran appears to be in the 2^nd^ and 3^rd^ decades of life in UC and CD patients, consistent with findings in the Western countries and Asia for CD. In contrast, UC occurred at a younger age range in the West and Asia(19). A bimodal age distribution of IBD development in Iranian patients was not observed, as it had been reported in the 6^th^ and 8^th^ decades in western populations(20). A delay has been shown in the diagnosis of some patients with UC, due to many factors including difficulty in diagnosis, infections colitis, lacking of awareness and follow-up.

Klement et al. from Australia reported an increased risk of inflammatory bowel disease in infants who were not breast-fed (21). Further study is also needed to confirm the increasing number of developing IBD in breast-fed patients during infancy.

A high incidence of a positive familial history of IBD among first and second-degree relatives in this study indicated a genetic role in pathogenesis of IBD similar to reports from western countries (22), and a study from Sri Lanka (23). Therefore, genetics plays an important role in the pathogenesis of IBD.

The exact relationship between genetic susceptibility and the role of the environment in the pathogenesis of IBD remain largely a mystery to researchers. Genetic susceptibility plays a key role in IBD development. Iranian patients with their different genetic reservoirs may demonstrate some novel characteristics for disease susceptibility.

C3435T polymorphism of the MDR1 gene has an association with UC in Iranian population as in western countries (24). A probable association of the Fok I polymorphism in the VDR receptor gene and Crohn's susceptibility in Iranian population was observed (25). CARD15/NOD2 gene was more frequent in CD patients than controls (26).

NOD2 exonic variations in Iranian Crohn's disease patients in 2011 were studied. Eight novel mutations were identified in the NOD2 exons, but the pathophysiological importance of these variants remains unclear (27). 

To advance our understanding of the key determinants of IBD in the developed and developing world, future population-based studies with a focus on reporting incidence and/or prevalence of IBD stratified by gene-environment-phenotype interactions seem necessary.

According to previous studies, smoking is the strongest environmental risk factor of CD (28). However, some nations with a high incidence of CD such as Canada and Sweden had a low prevalence of smoking in their populations. On the other hand, there is a high rate of smoking in Asian populations but a low incidence of CD (29). Thus, smoking needs to be further clarified in the population of this study. 

Firouzi et al. indicate that there is no association between appendectomy and development of CD and the protective effect in UC (30). Rates of appendectomies have decreased in developed countries, whereas the incidence of UC has remained constant. Moreover, the role of appendectomy in the development of CD has not been manifested in the West and Asia (31). Also, a decreased risk of developing UC due to appendectomy has been shown in the West, China and Japan (32, 33). This shows that appendectomy does not play a crucial role in the development of CD and UC.

Tonsillectomy was not associated with either UC or CD disease. The inverse association between ulcerative colitis and combined oral contraceptive pill (OCP) or non-steroidal anti-inflammatory drug (NSAID) in the Iranian population is noted (30). These findings of the current study are consistent with those of Andersson et al. (34). 

According to this study, diarrhea, hematochezia and bloody diarrhea seem to be the most common symptoms in UC patients. In comparison, the Chinese studies reported diarrhea, abdominal pain and bloody diarrhea as chief complaints of UC. Abdominal pain, diarrhea, weakness and weight loss were the predominant complaints in patients of CD patients, consistent with the reports in Chinese study (31).

The high incidence of extraintestinal manifestations in this study is similar to western countries and in contrast with other studies in Asia (35). According to a study from Boston, musculoskeletal disorders have been suggested the most common EIM in IBD patients (6). The knee was affected more than other joints in both diseases.

Low incidence of complications in this study differs from those in other studies from the West and Asia. Fistulating disease in patients with CD has been documented in a higher incidence than those reported from China (29). A low incidence of colorectal cancer was similar to reports from Asian countries (36), but careful follow-up in patients with extensive colitis lasting 8-10 years or more is needed.

The predominant form of UC was left-sided colitis and proctosigmoiditis, and exclusive proctitis was uncommon, consistent with reports from South Asian study (37). Assessing the location of CD in these studies shows that CD is confined to colon predominantly. The lack of data registry system was the biggest limitation in Iran. This study could provide a clue to a trend toward an increased prevalence of IBD in Iran. As IBD has an increasing trend during recent years, it seems beneficial to set up a control registry system to save patients’ data through the country. Since the etiology and pathogenesis of IBD are not fully understood, insight into the worldwide epidemiology of IBD is important to identify geographic patterns and time trends, highlight the burden of IBD globally, and determine possible environmental roles in IBD. As we move forward into the next two to three decades of IBD, we will face a series of challenges. We must:

1. Identify disease targets and patients who will benefit the most by certain classes of drugs.

2. Design treatment strategies that will decrease loss of response to therapy.

3. Identify individual risk factors for aggressive disease and treat those patients accordingly to change the natural history of disease.

4. Develop strategies that will minimize neoplastic and infectious risks of our targeted drug therapies.
